# Role of Layers and Neurons in Deep Learning With the Rectified Linear Unit

**DOI:** 10.7759/cureus.18866

**Published:** 2021-10-18

**Authors:** Akira Takekawa, Masayuki Kajiura, Hiroya Fukuda

**Affiliations:** 1 Graduate School of Frontier Science, Konan University, Kobe, JPN; 2 Graduate School of Human Development and Environment, Kobe University, Kobe, JPN

**Keywords:** rectified linear uniit (relu), role of neurons, role of layers, three types of irises, canonical correlation analysis, curved surface discrimination, deep learning

## Abstract

Deep learning is used to classify data into several groups based on nonlinear curved surfaces. In this paper, we focus on the theoretical analysis of deep learning using the rectified linear unit (ReLU) activation function. Because layers approximate a nonlinear curved surface, increasing the number of layers improves the approximation accuracy of the curved surface. While neurons perform a layer-by-layer approximation of the most appropriate hyperplanes, increasing their number cannot improve the results obtained via canonical correlation analysis (CCA). These results illustrate the functions of layers and neurons in deep learning with ReLU.

## Introduction

Deep learning classifier models are used to classify data into several groups. The classification is typically performed by inputting various features of the data through the classifier, which is typically composed of many neurons arranged in multiple layers [1]. The classifier passes each input through its various layers, and the final output value is used to classify each input. In each layer, except the input layer, each neuron passes its inputs through its activation function. The output of the activation function is then combined with an appropriate weight coefficient (weight) and passed to the next layer as input. The weights are optimized during model training using backpropagation. However, owing to a large number of weights and the complexity of the training process, it is difficult to clearly determine the role of each layer and neuron.

In this study, three types of irises [2]are classified based on features that include the properties of their calyxes and petals. Canonical correlation analysis (CCA), which is a method of multivariate analysis that classifies samples into multiple groups based on hyperplanes, and deep learning models are used for classification. The results of both methods are compared to determine the roles of the neurons and layers in the deep learning model using the rectified linear unit (ReLU). It is used as the activation function for the deep learning models. The ReLU function outputs zero when the input value is zero or negative and outputs the input value itself when the input is positive. This function emulates the firing behavior of nerve cells. Cybenko has also proved that using deep learning with the superpositions of a sigmoidal function can approximate any continuous function to an arbitrary accuracy [3]. By comparing the results of the CCA and deep learning in the classification of three types of irises, we confirm that any curved surface can be approximated by using deep learning with ReLU. Knowing the role of layers and neurons enables us to clearly understand the behavior of deep learning.

## Materials and methods

Deep learning model

**Figure 1:**
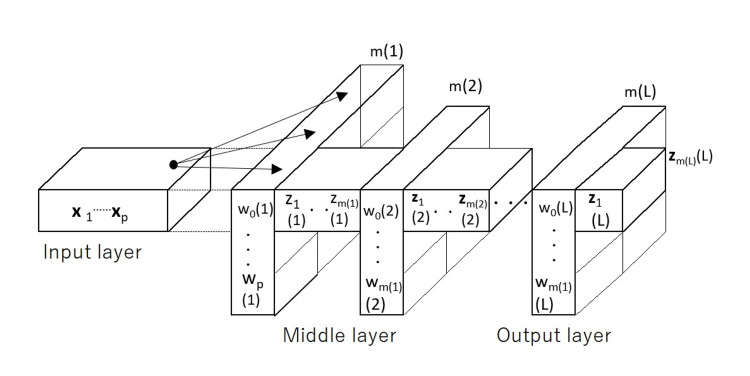
Deep learning model.

The deep learning model consists of three multi-overarching layers: the input, middle, and output layers. The input and output layers are always single layers, whereas the middle layer consists of multiple sublayers. The model is configured as shown in Figure 1.

The number of sample data is *n*, the feature quantity is **x**, and its dimension is *p*. The sample dataset is represented by ***x*** = (**x**_1_, **x**_2_, ･･･, **x**_p_). **x** is a matrix of *n* rows and *p* columns. The weight vector for the *k*th neuron in the first middle layer is **w**(*k*, 1) = {*w*_1_(*k*, 1), w_2_(*k*, 1), ･･･, w_p_(*k*, 1)}. The sum of **x*w***(*k*, 1)^T^ and constant bias *w*_0_(*k*, 1) is the total input to the *k*th neuron, where T represents the transposed matrix. We assume that the number of neurons in the first middle layer is *m*(1); then, the output vector, *z*(1), in the first middle layer is *z*(1) = {**xw**(k, 1)^T^ + *w*_0_(*k*, 1)}, *k* =1, ･･･, *m*(1). Next, **y**(1) is obtained by converting *z*(1) using the ReLU function. The output value, *z*(2), of the second middle layer, is obtained by multiplying **y**(1) and its layer weight, *w*(*k*, 2), and adding *w*_0_(*k*, 2). We assume that the number of neurons in the second middle layer is *m*(2); then **z**(2) = {**z**(1)*w*(*k*, 2)^T^ + *w*_0_(*k*, 2)}, *k* =1, ･･･, *m*(2).

**y**(2) is obtained by converting **z**(2) using the ReLU function. Because the ReLU function outputs zero when the input is nonpositive, we can assume the neuron does not exist. Therefore, it can be considered that **z**(1) = *y*(1) and *z*(2) = *y*(2). Then,** y**(2) =**y**(1)*w*(*k*, 2)^T^ + *w*_0_(*k*, 2), *k* =1, ･･･, *m*(2). This relationship holds for each sublayer of the middle layer. When **W**(ℓ+1)^T^ = (*w*(1, ℓ+1)^T^, ･･･, *w*(m(ℓ+1), ℓ+1)^T^) and *W*_0_(ℓ+1) = (*w*_0_(1), ℓ+1), ･･･, *w*_0_(*m*(ℓ+1), ℓ+1)), the relational expression between the ℓth layer and the (ℓ+1)th layer is

$$\mathbf{y}(ℓ+1) = \mathbf{y}(ℓ)\mathbf{W}(ℓ+1)^{T}+\mathbf{W}_{0}(ℓ+1)･･････ (1 )$$

If the output, **y**(*L*), of the final layer is expressed in terms of *x*, then

$$\mathbf{y}(L)=\mathbf{x}\prod_{i=1}^{L}\textbf{W}(i)^{T}+\sum_{k=0}^{L-1}\mathbf{W}_{0}(L-k)\prod_{i=L-k+1}^{L}\mathbf{W}(i)^{T} ･･････(2)$$

## Results

Role of layers

Takekawa et al. reported that curved surfaces can be approximated using multi-layered hyperplanes [4]. If there is a curved surface with *f* (**x**) = 0, then the curved surface is continuously differentiable in the range of **x**_0_ ≤ **x** ≤ **x**_L_, and when this range is divided into L equal intervals, (**x**_0_, *x*_1_, ･･･, *x*_L-1_), the curved surface for each of these intervals can be approximated by the tangent plane with respect to *f* (**x**) = 0 at each starting point. If L is increased and the interval is narrowed, the approximation accuracy of the curved surface is improved. Furthermore, there is a recurrence relationship between *f* (***x**_i_*) and *f *(***x**_i_*_ + 1_) : *f* (***x**_i_*_ + 1_) = *f* (**x**_i_) A*_i_* + B_i_, where *i*=0, ･･･, *L*-1. Here, A_i_ and B_*i*_are constants. The relationship of this recurrence formula is the same as that in Eq. (1). Thus, the layers in a deep learning model approximate a curved surface, and the degree of approximation to the curved surface increases as the number of layers increases. Therefore, we conclude that deep learning can discriminate between multiple groups based on a curved surface.

Role of neurons

Increasing the number of neurons has no effect on the approximation of the curved surface; it only increases the number of inputs to the hyperplane representing one layer. In CCA, an appropriate coefficient is applied to the input variable (explanatory variable), a linear transformation is performed, and a composite variable is created on the input side. In addition, appropriate coefficients are provided to the teacher data (objective variables) corresponding to the distinction between multiple groups, and a synthetic variable is created in the same way. CCA adjusts the coefficients to maximize the correlation between the two composite variables and enables the discrimination of multiple groups by the input variables. The first term on the right-hand side of Eq. (2) is the linear transformation of **x**. If we assume that deep learning maximizes the correlation coefficient between this value and the linear transformation of the teacher data, we can consider deep learning as a type of CCA. Therefore, increasing the number of neurons cannot improve the results of the CCA.

Verification based on the discrimination results of three types of irises

Here, we investigate the role of neurons and layers in deep learning by comparing the results of the CCA and deep learning discrimination for three types of irises [2]. The data used were the measured calyx length, calyx width, petal length, and petal width for 50 samples from each of the following members of the Iridaceae family: *Iris setosa* (group A), *Iris versicolor* (group B), and *Iris virginica* (group C). The true value and the classification result of the CCA were identical for groups A and C. However, 47 samples from group B were correctly classified, and three samples were incorrectly classified as group C, as shown in Table 1.

**Table 1:** Results of canonical correlation analysis.

Group	A	B	C
True	50	50	50
Classification	50	47	53

Next, the deep learning model with different numbers of sublayers in the middle layer (one sublayer, two sublayers, and three sublayers) was used to classify the sample dataset. Each of the sublayers had 15 neurons. The first and second models (one sublayer and two sublayers, respectively) were as accurate as the CCA. The number of neurons in each sublayer of the second model was increased to 20 and, later, to 30. However, the classification accuracy did not change. Finally, the third model with 15 neurons was equally accurate for all three groups, as shown in Table 2.

**Table 2:** Results of deep learning classification with three sublayers.

Group	A	B	C
True	50	50	50
Classification	50	50	50

## Discussion

The relationship between the layers of deep learning with ReLU as the activation function is represented by the recurrence formula of Eq. (1). When this recurrence formula is superposed in L layers, it can be seen that the result of superposition finally approximates one curved surface[4]. From this, it can be seen that deep learning is a discriminant analysis that discriminates the input data based on the curved surface and obtains the output categories. Here, increasing the number of layers corresponds to determining a curved surface that further improves the discrimination accuracy. Increasing the number of neurons simply improves the discriminant hyperplane accuracy, which only improves the CCA discrimination accuracy. Therefore, to obtain a result that outperforms the discrimination accuracy of CCA in deep learning, it is necessary to increase the number of layers of deep learning. We confirmed the certainty of this discussion by comparing the discrimination by CCA and discrimination by deep learning for three types of irises, with 50 samples each. Therefore, when A, B, and C, whose true types were known, were discriminated by CCA, all cases of A and C were correctly discriminated. However, for B, 47 cases were correctly discriminated, but three cases were incorrectly identified as C. When the same data were discriminated using deep learning, the discriminant results, when the intermediate layer was one layer and two layers, were the same as the CCA results. Therefore, the discrimination result when the number of neurons was increased from 15 to 20 or 30, while considering the middle layer as one layer, was the same as the discrimination result of CCA. The result was the same also when the intermediate layer was divided into two layers. When the number of neurons was maintained at the initial 15 and the number of layers was set to 3, the discrimination result was 100%, which was consistent with the true value. Thus, it can be seen that deep learning creates an optimally discriminated curved surface by increasing the number of layers and improves the discriminant accuracy.

## Conclusions

The roles of the layers and neurons in a deep learning model were discussed in this paper by means of a comparison with CCA. It can be concluded that deep learning distinguishes between groups based on a curved surface, and the layers approximate the curved surface. Moreover, increasing the number of layers improves the accuracy of the approximation. In contrast, increasing the number of neurons only improves the accuracy of the hyperplane approximation for each layer and not the approximation of the curved surfaces.
